# Notes and News

**Published:** 1900-04-07

**Authors:** 


### NOTES AND NEWS.

The Royal Meteorological Society has celebrated its jubilee this week, under the presidency of Dr. C. Theodore Williams.

The plague continues to prevail in Bombay and Calcutta. The practice as to inoculation in the two cities is very different. In Bombay upwards of 120,000 persons have been inoculated; whilst in Calcutta, with the exception of officials, the practice has hardly been followed at all.

We regret to see the announcement of the death of Dr. Robert Cory, well known as the director of the animal vaccine establishment in Lamb's Conduit Street, and vaccinator at the station of the Local Government Board in Blackfriars. His lectures on "The Theory and Practice of Vaccination," published about two years ago, contain a summary of the result of his long experience.

The importance of the sugar ration, especially for troops employed in warm climates, is generally recognised. A large quantity of what the Americans call "candy" has been sent to the United States troops in the Phillipine Islands, as well as to those in Cuba and Puerto Rico. The larger portion of these sweets are chocolate creams and lemon and other acidulated drops, consisting almost entirely of sugar and lemon or limejuice.

For some years the Maud Hospital at Exmouth was maintained by Mrs. Hume Long, who established it for the benefit of the district. At her death she left instructions in her will that the complete establishment should be offered to the local authorities within one year of her death. At a public meeting held at Exmouth a committee was appointed to take over the hospital on behalf of the town and neighbourhood, it being felt that the necessary income, amounting to about £450, would be forthcoming from those who recognised the benefits secured by the hospital. The hospital in future will be known as the Mrs. Hume Long Hospital.

Satisfactory results have followed upon the introduction of the open-air treatment at the Manchester Hospital for Consumption. In order that the City may be foremost in the ranks of those localities which are endeavouring to combat the disease, Mr. W. J. Crossley, the chairman of the Board of Management, will make the munificent gift of a new hospital to Manchester, situated in the Delamere Forest, to accommodate 100 patients, and costing about £60,000.

At Liverpool a milk vendor has been convicted of adding formalin to the milk sold by him, for purposes of preservation. His defence was that in the poor quarters where some of his customers resided the milk turned sour almost immediately unless some preservative were added. The vendor was required to enter into an undertaking to abandon the practice, and no penalty was exacted.

An unfortunate practitioner who has been much plagued by drunken although "qualified" assistants, is anxious that the General Medical Council should regard drunkenness amongst qualified assistants as "unprofessional conduct." The suggestion appears to be made in all seriousness, but, of course, what is sauce for the goose is sauce for the gander, and whatever might be the case as to assistants, we fancy there are a fair number of principals who would have considerable objection to any ruling which should deprive a practitioner of his diploma because under stress of weariness and pressure of hospitality he might be caught tripping. Let us be charitable; but let us at the same time be honest. The people who really are to blame are the amiable practitioners who, being thankful to get rid of an unsteady assistant at any price, smooth matters over by giving him a good character! We fancy these very broad-minded men are liable to legal process for so doing.

The arrival at Port Townsend, on Paget Sound, of a vessel from a Japanese port having on board nearly a score of cases of plague, and the occurrence in the heart of Chinatown, San Francisco, of a case of the disease the origin of which cannot be traced, give a serious interest to the question of plague in the United States. In hot climates the advent of hot weather generally brings some relaxation in the prevalence of this disease, but in temperate climates the opposite is apt to be the case, and when we consider the conditions of life in some of the more crowded American towns, we need not be surprised at the anxiety which is being expressed on the subject. Grounding our opinion on the history of epidemics as they have appeared in various places, it would seem that the single case in the midst of San Francisco is a more serious matter than the twenty on board ship at Port Townsend. These can be with considerable certainty isolated and rendered harmless, but a single untraceable case, occurring in a crowded city, and coming one knows not whence, has generally meant that the disease has really been going on for some time, and that far more widespread measures than the isolation of the individual must be taken if the disease is to be stopped. Among other things it has been decided that all sick Chinese shall be brought for treatment to white doctors, and that Chinatown shall be cleansed—which is good.

